# How Do Amusement, Anger and Fear Influence Heart Rate and Heart Rate Variability?

**DOI:** 10.3389/fnins.2019.01131

**Published:** 2019-10-18

**Authors:** Yan Wu, Ruolei Gu, Qiwei Yang, Yue-jia Luo

**Affiliations:** ^1^Sichuan Research Center of Applied Psychology, Chengdu Medical College, Chengdu, China; ^2^Key Laboratory of Behavioral Science, Institute of Psychology, Chinese Academy of Sciences, Beijing, China; ^3^Department of Psychology, University of Chinese Academy of Sciences, Beijing, China

**Keywords:** anger, fear, amusement, heart rate, heart rate variability, root mean square of successive differences

## Abstract

Emotions involve subjective experiences, behavioral performances, and physiological responses. Research concerning autonomic states corresponding to different emotions has prevailed for several decades. The present study was designed to investigate how specific emotions influence cardiac activities that reflect autonomic responses. Affective videos selected from a standardized Chinese database were used to induce amused, fearful, angry, and neutral emotions, while electrocardiogram and self-rated emotional experiences were recorded. Heart rate was significantly lower in the amused condition than in the angry, fearful and neutral condition. There were no significant differences among the latter three conditions. The root mean square of successive differences, an index of heart rate variability (HRV), was significantly larger in the amused condition than in the fearful, neutral, and angry conditions. It was also significantly larger in the angry condition than in the fearful condition. There were no significant differences between the fearful and neutral, or angry and neutral conditions. These results revealed that: (1) amusement activates the parasympathetic nervous system, and (2) compared with fear, anger is more likely to be linked with parasympathetic activation. We suggest that HRV, rather than the valence dimension (i.e., positive or negative) be regarded as a potential index to discriminate emotions related to approach or avoidance motivation.

## Introduction

As the most important entities that serve adaptive functions, emotions allow humans to behave in ways that increase their opportunities to survive and flourish (Lazarus, 1991). Emotional responses involve subjective experiences, behavioral, and physiological changes (Mauss et al., 2005). Research concerning the relation between subjective experience and physiological response dates back to the James-Lange theory of emotion, which suggested that the physiological response to a stimulus is antecedent to, and provides basis for, the emotional experience (Fehr and Stern, 1970). Although it is still debated in the scientific literature, this idea has led to a search for autonomic states specific to different emotions for several decades (Cacioppo et al., 2000). According to this theory, each kind of emotion expresses a person’s appraisal of a person-environment relationship involving a particular kind of harm or benefit. The appraisal generates action tendencies in response to the harm or benefit at hand. The preparation and execution of action sequences are emotion specific and supported by corresponding metabolic resources that are linked to the activity of the autonomic nervous system.

Heartbeat is primarily produced by the sinoatrial node, which generates action potentials that course throughout the cardiac tissue, causing regions of the heart muscles to contract in the orchestrated fashion that characterizes a heartbeat. Meanwhile, the heart is innervated by the sympathetic and parasympathetic branches of the autonomic nervous system, which regulate the heart rate by influencing the activity of the sinoatrial node. Activation of sympathetic fibers has an excitatory influence on the firing rate of the sinoatrial node, resulting in increased heart rate. Alternatively, the parasympathetic activation has an inhibitory influence on the pace-making activity of the sinoatrial node and produces decreased heart rate (Appelhans and Luecken, 2006).

In addition to the measurements of heart rate, the influence of the autonomic nervous system on the heart can also be indexed by heart rate variability (HRV), which refers to the beat-to-beat variation in heart rate. A growing body of psychological research supports an association between HRV and emotional responses (for a review see Appelhans and Luecken, 2006). Taking a step further, some researchers suggest that HRV could be an objective tool to assess emotional responses (Choi et al., 2017). According to the neurovisceral integration model proposed by Thayer and Lane (2000, 2009), HRV greatly indexes the inhibitory control of the prefrontal cortex over subcortical regions and the autonomic nervous system. HRV measures are derived by estimating the variation among a set of temporally ordered inter-beat intervals, which are mainly defined as the temporal distance between R-spikes (i.e., the prominent waveform that corresponds to the contraction of the heart’s ventricles). Variations in heart rate may be evaluated and categorized according to the statistical, frequency, and geometrical class of HRV (Camm et al., 1996). Among these three classes, statistical analyses are most frequently reported in previous studies and can be computed to represent overall HRV or HRV at different frequencies (Stein and Kleiger, 1999). Root mean square successive differences (RMSSD), as one of the measures calculated by statistical analyses, estimates short-term components of HRV (Camm et al., 1996). Since sympathetic influence on heart rate is mediated by neurotransmission of norepinephrine, variation of heart rate due to sympathetic activation emerges relatively slowly. For example, a peak effect is observed after about 4 s and the return to baseline occurs after about 20 s. On the contrary, the parasympathetic impact on the heart is mediated by acetylcholine neurotransmission and has a very short latency of response, with peak effect at about 0.5 s and return to baseline within 1 s (Pumprla et al., 2002). That is, compared to the sympathetic nervous system, the activity of the parasympathetic nervous system is mainly related to short-term components of HRV, which can be indexed by the RMSSD.

To explore the pattern of physiological activities associated with specific emotions, which are usually differentiated by subjective emotional experience, a variety of emotions should be elicited and manipulated during the experiment. Many researchers have used the International Affective Picture System as a standard tool of emotion simulation (Lane et al., 2000; Choi et al., 2017). The International Affective Picture System has the advantage of providing a standardized stimulus set to assess human emotions along the dimensions of valence (positive or negative) and arousal (Lang et al., 1999). In the literature on autonomic correlates of emotion, many investigators have reported reliable relations between the arousal and magnitude of autonomic output. This evidence suggests that the most consistent correlate of autonomic discharge is the arousal (or intensity) dimension of emotion. In contrast, there are no replicable findings that distinguish between positive and negative affect (Watson et al., 1999). To explain this phenomenon, we argue that the valence dimension of emotion fails to capture the characteristics of autonomic nervous system activity associated with specific emotions. For example, although both anger and fear belong to the same valence (i.e., negative emotion), they have a completely different impact on motivation (Lerner and Keltner, 2001; Carver and Harmon-Jones, 2009). Therefore, one should differentiate between different kinds of specific emotions regardless of whether they belong to the same valence category. Hence, the aim of this study was to identify the types of physiological patterns associated with specific emotions elicited by video clips that were selected from the standardized Chinese affective video (CAVS) (see section “Materials and Methods” for more details).

We would contrast anger with fear, both of which belong to negative emotions, to discern whether motivation influences HR or HRV. Meanwhile, these negative emotions would also be contrasted with positive emotion to find the influence of emotional valence on HR and HRV. Previous research has revealed that most of the negative emotions elicited by various means increased HR and decreased HRV, while positive emotion had an ambivalent influence on HR and HRV (Kreibig, 2010). Therefore, we expected that amusement would decrease HR and increase HRV when compared with fear and anger. As anger is related with approach motivation and fear with avoidance motivation, anger was expected to increase HRV when compared with fear.

## Materials and Methods

### Participants

Thirty-two right-handed students (15 women; mean age = 19.8 ± 1.4 years) from Chengdu Medical College participated in the experiment. All participants were free of neurological and psychiatric disorders, and had normal or corrected-to-normal vision. They provided their informed consent prior to the experiment. The local ethics committee approved the experimental protocol.

### Stimuli and Procedure

Video clips were used in this experiment to induce angry, fearful, amused, and neutral emotions. These video clips were selected from the standardized Chinese affective video system (CAVS), which was developed at the National Key Laboratory of Cognitive Neuroscience and Learning, Beijing Normal University. The CAVS includes thirty video clips, which belong to six categories (fearful, amused, sad, angry, disgusted, surprised, and neutral), with each category containing five video clips. Fifty Chinese college students rated these video clips with respect to differentiation, valence and arousal. The pretest for this system showed that the CAVS is reliable across Chinese individuals in emotional inducement (the intensity of target emotion is significantly stronger than others for all the six categories). More details about the CAVS are accessible in a previous report by Xu et al. (2010).

According to their differentiation level, we selected three video clips from each of the amused, fearful, and angry categories. The duration of each video clip ranged between 1 and 3 min. The order of the presentation of the three categories of emotion was counterbalanced across participants, and the three video clips of the same category were presented one by one. In other word, once the order of the presentation of the three specific emotions was determined for each participant, three video clips of one specific emotion would be presented one by one. Between the presentations of every two categories of video clips, a neutral video was presented; thus there were two neutral video clips in total in the experiment. Immediately following the presentation of each video clip, participants were required to evaluate their emotional experience associated with that video clip by selecting from four options: “amused,” “fearful,” “angry,” and “others.” Then the emotion arousal was rated on a 7-point scale.

The stimuli were presented and behavioral responses were collected using E-Prime (Version 2.0, PST, Inc., Pittsburgh, PA, United States). After the experiment, the participants were paid 30 Yuan RMB (approximately five dollars) for remuneration.

### Electrocardiogram (ECG) Recording and Analysis

Electrocardiogram was recorded from two extended electrodes of eego MyLab (ANT Neuro, Dutch), which were placed on the left wrist and ankle. The ECG was amplified using a 0.01–100 Hz band-pass and continuously sampled at 1000 Hz. The off-line analysis was conducted in MATLAB R2013b (Mathworks, Natick, MA, United States). On a typical ECG, the main spike is the QRS complex, which is the combination of the Q, R, and S waves occurring in rapid succession. The QRS complex reflects a single event of the depolarization of the right and left ventricles of the human heart; thus, they are usually considered together. To detect the spike of the R wave in each QRS complex, we used the following algorithm. When the voltage difference between two time points with an interval of 25 milliseconds is larger than 500 microvolts, the maximum value within the interval from the former time point up to the one 55 milliseconds later is taken as the spike of the R wave. We selected the last minute of the video clip that elicited the expected emotion with maximum strength according to the evaluation of each participant from the three ones for ECG analysis for each emotion category. In the current study, RMSSD was used as an index of HRV (Camm et al., 1996).

Statistical analysis was performed using SPSS (17.0; SPSS, Inc., Chicago, IL, United States). The *p*-values of all the main effects and interactions were corrected by applying the Greenhouse-Geisser method when needed. *Post hoc* testing of significant main effects was conducted with Bonferroni correction. Meanwhile, the least significant difference (LSD) method also was used to decrease the probability of making Type II Error when needed. Significant interactions were further examined using simple-effect analysis. Partial eta-squared (η*_*p*_^2^*) values were reported to examine the size of effects in ANOVA models, where 0.05 represented a small effect, 0.1 represented a medium effect, and 0.2 represented a large effect (Cohen, 1973).

## Results

### Emotional Experience

**FIGURE 1:**
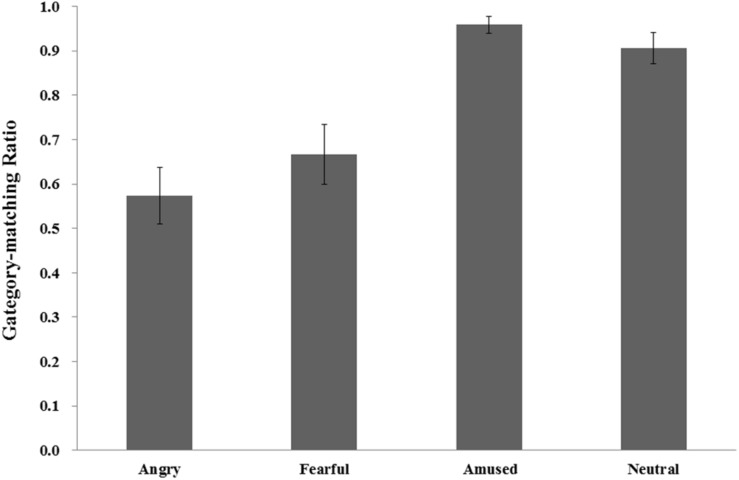
The category-matching ratios (i.e., the ratio of the number of participants’ choices that matched the emotion category of a video clip to the total number of video clips in the emotion category).

The participants’ emotional experiences associated with the affective video clips were evaluated in two aspects: category and arousal. First, the category-matching ratios (i.e., the ratio of the number of participants’ choices that matched the emotion category of a video clip to the total number of video clips in the emotion category) were 0.57, 0.67, 0.96, and 0.91 for angry, fearful, amused and neutral video clips, respectively, [*F*(2.21, 68.56) = 16.83, *p* < 0.001, η*_*p*_^2^* = 0.35]. A *post hoc* test with Bonferroni correction revealed that the category-matching ratios of amused and neutral video clips were significantly higher than those of angry and fearful video clips (all *p*s < 0.001), but neither the difference between the former two conditions nor the difference between the latter two conditions were significant (all *p*s > 0.05) (see Figure 1).

**FIGURE 2:**
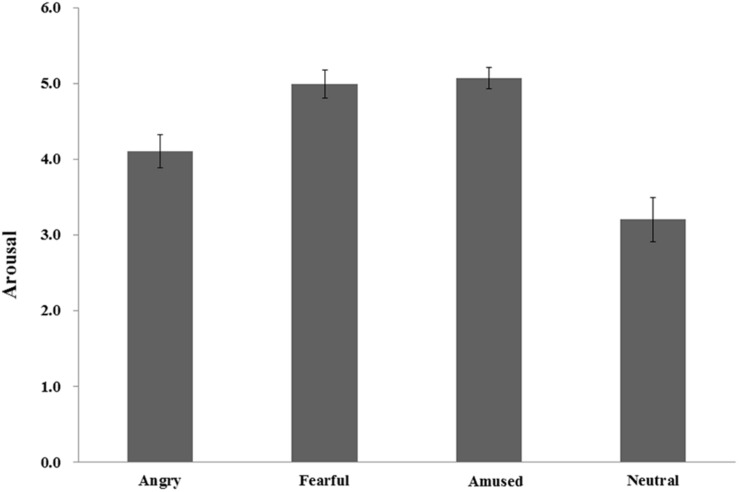
The arousal level of emotional experience.

The arousal level of emotional experience was significantly different between angry (4.10), fearful (4.99), amused (5.07), and neutral (3.20) video clips [*F*(2.42, 74.91) = 19.24, *p* < 0.001, η*_*p*_^2^* = 0.38]. A *post hoc* test with Bonferroni correction showed that the arousal level of angry and neutral video clips were significantly lower than that of fearful and amused clips (all *p*s < 0.05), but neither the difference between the former two conditions nor the difference between the latter two conditions were significant (*p* > 0.05) (see Figure 2).

### ECG Results

**FIGURE 3:**
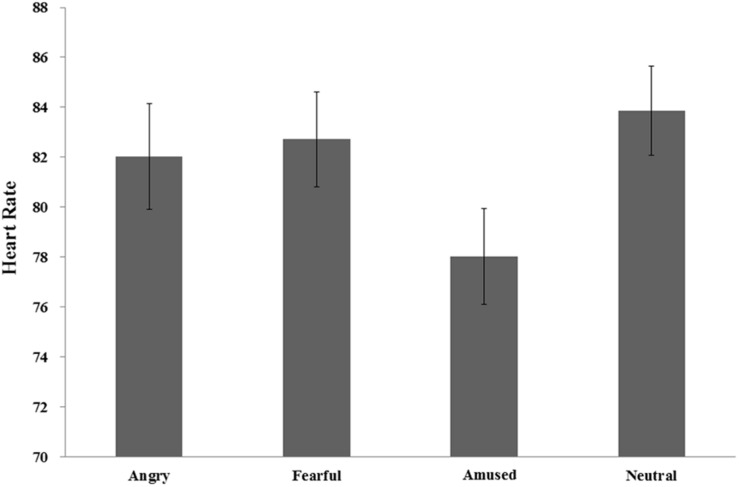
Heart rate.

Heart rate was significantly different between the four emotional conditions [*F*(2.48, 76.90) = 5.38, *p* = 0.004, η*_*p*_^2^* = 0.15]. A *post hoc* test with Bonferroni correction showed that the differences of heart rate were not significant among the angry (82.03), fearful (82.73), amused (78.03) and neutral (83.88) conditions (all *p*s > 0.05), except the difference between the latter two condition (*p* < 0.05). A *post hoc* LSD test with arousal as covariate showed that the heart rate was significantly lower in the amused condition than in the angry, fearful and neutral conditions (all *p*s < 0.05). There were no significant differences among the latter three conditions (all *p*s > 0.05) (see Figure 3).

**FIGURE 4:**
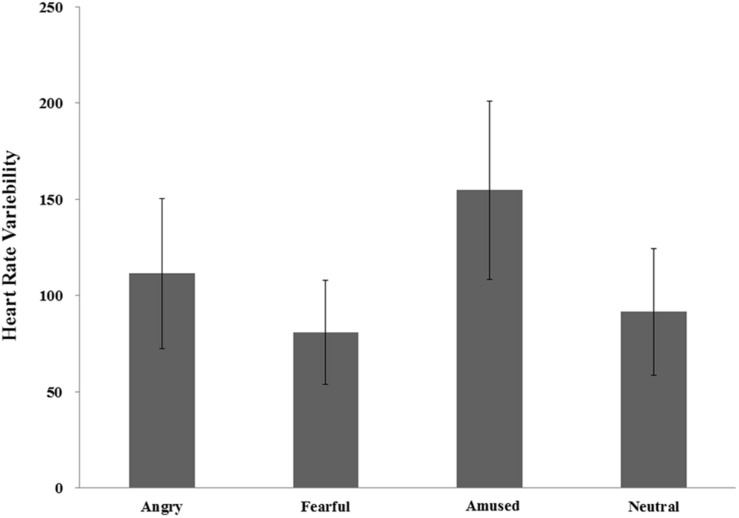
Heart rate variability (HRV).

RMSSD was significantly different between the four conditions [*F*(2, 62) = 5.47, *p* < 0.01, η*_*p*_^2^* = 0.15]. A *post hoc* test with Bonferroni correction showed that the differences of RMSSD were not significant among the amused (154.74 milliseconds), fearful (80.81 milliseconds), angry (111.46 milliseconds) and neutral (91.42 milliseconds) conditions (all *p*s > 0.05), except the difference between the former two condition (*p* < 0.05). A *post hoc* LSD test with arousal as covariate showed that the RMSSD was significantly higher in the amused condition than in the fearful, neutral (all *p*s > 0.05), and angry conditions (*p* = 0.052). The RMSSD was also significantly higher in the angry condition than in the fearful condition (*p* < 0.01). There were no significant differences between the fearful and the neutral conditions (*p* = 0.49), between the amused and the angry conditions (*p* = 0.42), or between the angry and the neutral conditions (*p* = 0.15) (see Figure 4).

## Discussion

More than one hundred years ago, William James maintained that discrete emotional experiences could be identified with unique patterns of bodily changes, which are produced by autonomic nervous system activity. This notion was critiqued by Cannon (1927), who provided evidence that autonomic events are too slow, intensive, and undifferentiated to contribute to emotions. However, recent investigations have suggested that the patterns of autonomic activities could be related to discrete emotions, supporting James’s original idea (Cacioppo et al., 2000).

Findings from the early research on autonomic activity appeared to be heterogeneous. According to Levenson (1988), this inconsistency in prior studies was largely due to various methodological problems, including the failure to identify that an independent emotional state has been aroused and the unreliability of physiological recordings. To solve these problems, we selected the three most differentiated video clips for amused, fearful, and angry inducement, respectively, from the CAVS, which contains standardized video for specific emotional evoking. Further, the participants were required to evaluate their subjective emotional experience to each video clip immediately after watching it to ensure reliability of their appraisals. Additionally, autonomic analysis was done while participants viewed the video clips as the intensity of each emotion was expected to be the highest during this time. With these experimental manipulations, we expected that specific emotions elicited for the analysis of autonomic activity would be consistent with self-reported ratings.

In the present study, we investigated the influence of specific emotions on the pattern of cardiac activities, and found that amusement led to a decreased heart rate. This result is consistent with a meta-analysis that revealed that the heart rate is lower for amusement than that of fear, anger, and neutral emotions (Cacioppo et al., 1997; Kreibig, 2010). The current results also revealed that there was no significant difference in heart rate between anger (or fear) and the neutral category of emotion. In this study, we required the participants to watch affective movies to elicit emotions. Presumably, watching movies safely in the laboratory may have weakened the physiological responses of the participants in the anger and fear conditions. Consequently, this study did not find that anger and fear accelerate heart rate, as reported in previous studies (Cacioppo et al., 2000). Further research is therefore needed to investigate whether experienced anger and fear do in fact accelerate heart rate.

More importantly, the results showed that the HRV was larger in the amusement than anger, fear, and neutral conditions. HRV is related to the activity of the parasympathetic nervous system. Considering the decreased heart rate for amusement, we can infer that amusement selectively activates the parasympathetic nervous system. Since the parasympathetic nervous system and the sympathetic nervous system antagonize each other, the parasympathetic activation inhibits the sympathetic activity. When we are amused, we feel relaxed and contented. This feeling is also regarded as a feature of the parasympathetic activation and the sympathetic inhibition, which helps an organism to reserve mental and physiological resource (Porges, 2001). The results also revealed that HRV was larger in the anger than the fear condition. Although both anger and fear belong to negative emotions, anger is often linked to approach motivation, while fear is linked to avoidance motivation or freezing up (Harmon-Jones et al., 2013). The current results suggest that HRV, which is linked to the autonomic activities, perhaps reflects approach motivation to some extent, i.e., low parasympathetic activities coupled with uninhibited sympathetic activities induced by emotions, such as fear, and indexed by low HRV, prompt avoidance, while moderate parasympathetic with moderate sympathetic activities induced by emotions, such anger, and indexed by medium HRV, prompt approach. As for emotions with parasympathetic activation coupled with sympathetic inhibition, such as amusement or relaxation, motivations of both approach and avoidance decrease.

Limitations still exist in the present study. First, the *Post hoc* testing of RMSSD was based on the LSD method. Although this method has the advantage of decreasing the probability of making Type II Error, it increases the probability of making Type I Error. When the Bonferroni correction was applied to the *post hoc* test of RMSSD, the difference between the amused and angry conditions, the difference between the amused and neutral conditions, and the difference between the angry and fearful conditions were not significant. Further research is still needed to clarify if this result was caused by the reason of small sample. Second, the video clips used in this study were selected from the CAVS, which contains six emotion categories. In the six categories, only one category contains a positive emotion, labeled as “
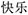
” in Chinese or “happiness” in English. The video clips in this category are all comedies, which actually elicit the emotion of amusement. Other positive emotions with similar meanings, such as happiness and contentment, are more strongly elicited by real life events than through watching affective videos. However, these emotions have different influences on autonomic nervous system activity (Kreibig, 2010). Therefore, the ECG activity of amusement in this study could not be extended to other similar emotions.

To sum up, amusement elicited by watching video clips led to a decrease in heart rate and increase in HRV, when compared with angry, fearful, and neutral emotions. This suggests that amusement is related to the activation of the parasympathetic nervous system. Furthermore, anger led to increased HRV when compared with fear, suggesting that HRV could discriminate certain kinds of negative emotions. These results indicate that amusement, anger and fear can be differentiated by the ECG signals, contributing to the literature that objectively measures subjective experiences of emotions. Further research is still needed to differentiate between the peripheral physiologies of other specific emotions.

## Data Availability Statement

The datasets generated for this study are available on request to the corresponding author.

## Ethics Statement

The studies involving human participants were reviewed and approved by the ethics committee of Chengdu Medical College. The patients/participants provided their written informed consent to participate in this study.

## Author Contributions

YW and QY conceived and designed the experiments. YW performed the experiments. QY analyzed the data. QY and RG wrote the manuscript. Y-JL contributed to the materials and analysis tools.

## Conflict of Interest

The authors declare that the research was conducted in the absence of any commercial or financial relationships that could be construed as a potential conflict of interest.
